# Are Wealthier Times Healthier in Cities? Economic Fluctuations and Mortality in Urban Areas of Latin America

**DOI:** 10.3389/ijph.2021.1604318

**Published:** 2021-12-09

**Authors:** Carlos Marcelo Leveau, José A. Tapia Granados, Maria Izabel Dos Santos, Marianela Castillo-Riquelme, Marcio Alazraqui

**Affiliations:** 1 Consejo Nacional de Investigaciones Científicas y Técnicas (CONICET), Buenos Aires, Argentina; 2 Instituto de Producción, Economía y Trabajo (IPET), Universidad Nacional de Lanús, Remedios de Escalada, Argentina; 3 Department of Politics, Drexel University, Philadelphia, PA, United States; 4 Centro de Integração de Dados e Conhecimentos para a Saúde (CIDACS), Fundação Oswaldo Cruz, Salvador de Bahía, Brazil; 5 Escuela de Salud Pública, Universidad de Chile, Santiago, Chile; 6 Instituto de Salud Colectiva, Universidad Nacional de Lanús, Remedios de Escalada, Argentina

**Keywords:** mortality, health economics, economic recession, cities, Latin America

## Abstract

**Objective:**

To analyze the relationship between economic conditions and mortality in cities of Latin America.

**Methods:**

We analyzed data from 340 urban areas in ten countries: Argentina, Brazil, Chile, Colombia, Costa Rica, Guatemala, Mexico, Panama, Peru, and El Salvador. We used panel models adjusted for space-invariant and time-invariant factors to examine whether changes in area gross domestic product (GDP) per capita were associated with changes in mortality.

**Results:**

We find procyclical oscillations in mortality (i.e., higher mortality with higher GDP per capita) for total mortality, female population, populations of 0–9 and 45+ years, mortality due to cardiovascular diseases, malignant neoplasms, diabetes mellitus, respiratory infections and road traffic injuries. Homicides appear countercyclical, with higher levels at lower GDP per capita.

**Conclusions:**

Our results reveal large heterogeneity, but in our sample of cities, for specific population groups and causes of death, mortality oscillates procyclically, increasing when GDP per capita increases. In contrast we find few instances of countercyclical mortality.

## Introduction

There appear to be differences between high-income and low/middle-income countries regarding how mortality evolves during business cycles of expansion and recession. While procyclical oscillations predominate in the first group of countries [1–15], in the second group of countries there is no clear relationship between the economy and mortality. Using data from countries in Latin America, both countercyclical [16–20] and procyclical [21, 22] oscillations of mortality have been reported, while some studies have yielded conflicting results [16] or have found no association between the business cycle and mortality [17].

Latin American countries have suffered major economic disturbances during recent decades, including the debt crises of the 1980s, the financial crises of the 1990s (the *tequila* crisis in Mexico), the recessions around the turn of the century with financial collapse of Argentina in 2001 (the *corralito*), the regional impact of the global recession in 2009 and the current economic crisis triggered by the COVID-19 pandemic. These crises are mostly felt in the urban areas where 80% of the Latin American population lives [23]. In addition, Latin America is characterized by large social inequalities, and these inequalities are also present across geographic areas including cities [24]. Within each Latin American country, cities with living conditions similar to developed countries coexist with cities with the worst living conditions for a developing country. These territorial inequalities may imply different relationships between the economy and mortality. In Mexico countercyclical mortality was found in states of low socioeconomic development while mortality fluctuated procyclically in states of higher socioeconomic development [22].

Economic growth might reduce mortality in low socioeconomic level areas by allowing greater access to healthpromoting goods and services [22, 25]. It could however increase mortality by allowing more consumption of noxious substances, decreasing sleep time or leisure time and also increasing the exposure to harmful levels of pollution, traffic, stress in the work environment, etc.

Most investigations of business-cycle effects on mortality have focused on a single country and, to our knowledge, only one study focused on cities, specifically US metropolitan areas [11]. Our study is the first investigation on the effect of macroeconomic variations on mortality in urban areas of low/middle income countries. The aims of the study are: 1) to assess the association between mortality and regional economic conditions in Latin American cities; and 2) to assess whether this association is modified by the socioeconomic level of the cities.

## Methods

This investigation used data from the SALURBAL study [26], a data platform for cross-country studies of urban health that integrates data on indicators of health and the physical and social environment of the urban areas of eleven Latin American countries. We analyzed data from 340 urban areas or “cities,” each one with a population of at least 100,000 inhabitants in 2010 in ten countries: Argentina, Brazil, Chile, Colombia, Costa Rica, Guatemala, Mexico, Panama, Peru, and El Salvador (see details in Supplementary Table A1, Supplementary material). These cities represent more than half of the total population of each country, except in El Salvador (30%), Guatemala (24%) and Peru (47%). Nicaragua is included in SALURBAL but proper mortality data were not available so Nicaragua was not included in this study. City limits were defined based on political or administrative boundaries as a single administrative unit (e.g., *municipio*) or as a combination of adjacent administrative units (e.g., several *municipios*) that are part of the urban extent as determined from satellite imagery [26]. The sample period was determined by the availability of mortality and gross domestic product (GDP) data (Supplementary Table A1).

All-cause and cause-specific mortality rates were calculated by sex and age groups (0–9, 10–29, 30–44, 45–64, 65+ years). Causespecific mortality was calculated for the seven major causes of death—cardiovascular disease (CVD), respiratory diseases, malignant neoplasms, diabetes mellitus, respiratory infections, infectious and parasitic diseases, nutritional deficiencies—, and for three qualitatively important causes—road traffic injuries, suicide, and homicide. ICD9 and ICD10 codes were grouped in categories using the World Health Organization Global Health Estimates classification [27]. More details on ICD codes are available in Supplementary Table A2 (Supplementary material). The years with available mortality data varied by country: for Argentina 2010–2015, for Brazil 2002–2015, for Chile 2004–2015, for Colombia 1990–2015, for Costa Rica 2010–2015, for Guatemala 2009–2015, for El Salvador 2010–2014, for Mexico 2005–2015, for Peru 2008–2015, and for Panama 2012–2015.

Three common concerns with vital registration data in low- and middle-income countries are 1) missing information on sex and age, 2) deaths coded as ill-defined diseases or injuries of ill-defined intent, and 3) underregistration. The proportion of ill-defined causes of death was below 5% in Colombia, Costa Rica, Mexico, Panama, and Peru; between 5 and 10% in Argentina, Brazil, and Guatemala; nearly 20% in El Salvador in the last available year. The completeness of the death registration was above 90% in Argentina, Brazil, Chile, Guatemala, and Panama; between 76 and 87% in Colombia, Costa Rica, and Salvador; and 62% in Peru [28]. To address these issues, single conditional imputation was applied for 1) imputing sex in each record with missing sex using the observed proportion of males in the same age (5-years groups)/cause of death strata for each country-year. The same procedure was used for imputing age using sex and cause of death. Regarding 2), for all deaths by an ill-defined disease/intent in each 5-years age group, sex, country and year, we assigned a cause of death using a single multinomial draw, based on observed probabilities of causes of death. The completeness of death registration was estimated using a set of demographic methods and incorporated in calculating mortality rates for 3). These methods check whether the number of deaths registered matches what would be expected based on the age distribution between two time points (generally two censuses). We used three different methods: generalized growth balance, synthetic extinct generations, and the hybrid method. Additional details on each procedure are available elsewhere [29]. In order to estimate total mortality rates and mortality rates by sex, age group, and cause of death, estimated and projected population counts were obtained from the statistical agencies of each country.

GDP per capita data used in the analysis were obtained from Kummu et al. [30] and cover the years 1990–2015. This database contains annual subnational GDP per capita (i.e., province, state, department, region, etc.; national level only for Costa Rica) that we assigned in this investigation to 366 cities of 10 countries. GDP amounts are in US dollars of 2011 obtained by conversion of local currencies into dollars using purchasing power parities (PPP). Additional details on methods and sources are available elsewhere [30]. For 26 cities we observed an annual change of 50% or more in absolute terms at some point over the study period. These cities (355 observations) were dropped from the analytical sample becauseGDP values were judged unreliable. To characterize cities according to their socioeconomic status (SES), we divided the cities into tertiles according to the GDP per capita of 2014. This was the last year with data of GDP per capita in all the cities.

We used panel regressions to study the short-term associations between economic conditions and mortality. The annual mortality rate (per 100,000 inhabitants) was the dependent variable and GDP per capita was the main explanatory variable. The main model is:

(1) $$M_{ct}=\alpha +\beta G_{ct}+\Pi_{t}+\Omega_{c}+\gamma D_{ct}+{\text{λ}}_{c}t+\varepsilon_{ct}$$

where *M_ct_* is a measure of mortality for city *c* in year *t*; *G_ct_* is citylevel GDP per capita; *Π_t_* and *Ω_c_* are respectively fixed effects for year and city; *D_ct_* is a vector of city demographic controls (percentage of population under 10 years of age; percentage of population aged 65 or over; and a masculinity index); λ*_c_t* is a cityspecific linear time trend; and *ε_ct_* is the error term.

In addition to the main model (Model 1, M1), five other models were considered: Model 2 (M2) is a model including the time-varying variables after subtracting trends computed via a Hodrick–Prescott (HP) filter with smoothing parameter *λ* = 100 [4]. Model 3 (M3) is like M1 but with 1-year lag in GDP per capita. Model 4 (M4) is similar to M3 but included time-varying variables after subtracting trends computed via a HP filter with smoothing parameter *λ* = 100. Model 5 (M5) test the differential effect of macroeconomic variations on mortality according to the socioeconomic level of the cities. To do this, we used M1 but replacing the term *βG_ct_* by βG_ct_*S_ct_, where *S_ct_* is a categorization in tertiles of the socioeconomic city-level score. M5 also included interactions between *S_ct_* and the other covariates in the regression. Finally, Model 6 (M6) is similar to M5 but included the timevarying variables after subtracting trends computed via a HP filter with smoothing parameter *λ* = 100.

All regression models were weighted by the square root of the city population to account for heteroscedasticity and standard errors were clustered by city. City-specific fixed effects and linear trends are unneeded in M2, M4 and M6 in which detrending is effected by the HP filter (Ionides et al. 2013). Models with sex-specific death rates included sex-specific percentages of child population (age below 10) and advanced-age population (ages 65 and over) as covariates. Models with age-specific mortality rates as dependent variable included a masculinity index computed as the ratio of men to women in the specific population. All analyses were conducted in Stata version 13.1 (StataCorp, College Station, TX).

The aim of this study was to use newly available data on mortality and GDP for urban areas of Latin America to study the impact of economic fluctuations on mortality. We used several slightly different panel models that are usually considered as proper procedures to analyze this issue. We present here the results of various models to assess the robustness of the results to the concrete method of analysis. In our research there was not any shifting of focus or altering of the methods once the data were being examined.

## Results

Table 1 shows the descriptive statistics of the analyzed variables. As expected, mortality averages and measures of variability exponentially increase with age and mortality in men is higher than mortality in women. CVD and malignant neoplasms were the leading causes of death. On average, cities had a higher proportion of female population, with a greater contribution from the elderly population compared to the young population.

### All Cities and Analysis by Country

Table 2 presents results of models M1 and M2. Considering the different outcomes, statistical models and the different samples we investigated, mortality appeared mostly procyclical, with positive and statistically significant effects of GDP per capita on total mortality (though only in M2), death rates for many age strata, causes of deaths, and female mortality. Considering specific countries, there were procyclical oscillations of total mortality, in Colombia (though only in M1), Chile (though only in M2) and Peru. Mortality of women appeared procyclical in the whole sample and in Brazil, Colombia, Chile and Peru. Results for age-specific mortality showed procyclical oscillations in children below age 10, as well as in adults aged 45–64, and 65+. These procyclical associations appeared also in Argentina (45–64 years in M2), Brazil (65+ years), Chile (45–64 years), and Colombia (0–9 years in M1).

Analyses of cause-specific mortality in all cities showed economic expansion positively associated with mortality due to CVD, malignant neoplasms, diabetes mellitus, respiratory infections, and road traffic injuries (Table 2). Homicides are the only cause-specific mortality that we found increasing countercyclically in recessions in most models and samples, though only one result of the model M2 for the sample including all cities is statistically significant.

### Analysis by Tertiles of SES

Table 3 shows the results of analyzing all cities stratified by tertiles of SES. Results for total mortality were procyclical in all tertiles, though not statistically significant. Female mortality appeared procyclical in the tertile of medium SES. Results for age-specific mortality revealed procyclical oscillations mostly in the tertiles of medium and high SES (Table 3). Results for specific causes showed procyclical mortality mostly in the tertile of medium SES. Homicides (in M2) are the only cause-specific mortality that appears countercyclical in the tertile of medium SES.

### Lagged Effects and Robustness of Results

Results from models M3 and M4 with 1-year lagged GDP per capita showed mostly the same results compared with models M1 and M2 (Supplementary Table A3 in Supplementary material). With the exception of malignant neoplasms, the statistically significant coefficients in models M1 and M2 did not show sign changes in models M3 and M4. Considering specific countries the statistically significant coefficients in M1 and M2 mostly did not show sign changes in M3 and M4.

We also computed models applying different restrictions to balance the panel data. First, we restricted observations to the period 2002–2015 in Colombia (Supplementary Table A4 in Supplementary material). Results of these models were largely unaffected, except for total mortality (statistically not significant in M2), population aged 10–29 years (statistically significant in both models), and homicides (statistically significant in M1). Second, we exclude countries with less than 20 observations and obtained similar results to those in Table 2 (Supplementary Table A5 in Supplementary material). Third, we excluded the observations from Brazilian cities—41% of all observations (Supplementary Table A5 in Supplementary material)— and obtained results mostly similar, except for total mortality (statistically not significant), diabetes mellitus (now statistically not significant), infectious and parasitic diseases (now statistically significant in M2), nutritional deficiencies (statistically significant in M2), and homicides (statistically not significant).

## Discussion

To our knowledge, this is the first study to analyze the association between economic cycles and mortality in cities of developing countries. Former studies using data from Latin American countries have shown acyclical or countercyclical oscillations of mortality [17–19], although one study with Mexican data showed a procyclical oscillation [22] and an Argentinian investigation showed inconclusive results [16]. We found positive and significant effects of GDP per capita on total mortality—i.e., procyclical mortality—for all cities, in Chile, Colombia and Peru; in Mexico, Brazil and Argentina we found effects that are non-significantly different from zero—which can be interpreted as acyclical mortality.

Stratified analyses by sex reveal procyclical mortality of women in the general sample and in most subsamples. Women have a greater participation in the informal economies of urban Latin America compared to men [31] and, furthermore, Latin American women face lower rates of labor participation and higher unemployment rates than men [32]. These three factors may explain the fact that women seems in our analysis more vulnerable to the fluctuations of economic growth. According to Seguino [33], if economic growth occurs in a context of economic liberalization, women are more vulnerable than men to economic insecurity and labor flexibility, which would reduce the mortality ratio between women and men during the economic expansion. On the other hand, the acyclic association between male mortality and business cycles may perhaps be explained by the opposite patterns of mortality due to chronic diseases and transport injuries (procyclical oscillations) and homicides (countercyclical oscillation). Interpersonal violence is a more serious public health problem in Latin America than in high-income countries. For example, in our sample of cities the homicide rate is higher than the death rate from transport injuries, while the opposite is the case in US cities [11].

The procyclical oscillation of child mortality (0–9 years) found in our study could be caused by traffic deaths, since traffic mortality is strongly procyclical [9] and other studies [34, 35] found decreases in traffic deaths in children during the economic crisis of 2007–2008. As the workforce increases due to economic growth, the ability of parents to provide care for their children is reduced, probably increasing their risk of death from traffic injury and other unintentional injuries. Considering the data analyzed in our sample of cities, mortality from unintentional injuries in children aged 0–9 years is the 3rd most frequent cause of death in Argentina, Chile and Mexico, the 4th most frequent in Brazil and Peru, and the 5th most frequent in Colombia. Alcohol consumption could increase during periods of economic growth [36], which could affect the effectiveness of supervision to protect children from harm [37].

Our results show procyclical oscillations in mortality due to CVD, malignant neoplasms, diabetes mellitus, respiratory infections and transport injuries, and a countercyclical oscillation of homicides. Regarding mortality from CVD and respiratory infections, economic growth may be associated with air pollution [38] and its impact would be greater in cities due to the high geographic concentration of economic activity. In addition, motorization, which accelerates in times of economic growth, is the most important contributor to atmospheric pollution in Latin American cities [39] and increases the probability of vehicle crash, pedestrian or cyclist hit and traffic deaths overall. Procyclical traffic mortality has been found without exception in both developed countries [2–4, 7–10, 12, 14] and in Latin America [16, 22]. Regarding cancer, its procyclical oscillation similar to other deaths from chronic diseases suggests the presence of similar causal mechanisms related to the adoption of healthier habits—e.g., less consumption of alcohol and tobacco—during periods of recession [40]. In addition, other diseases like respiratory infections can increase the risk of death of individuals with a malignancy that will be considered the cause of death [41].

Homicides showed a countercyclical oscillation in our samples. Our findings contrast with two studies in Mexico showing procyclical homicide rates [21, 22], while other studies in Latin America reported no connection of homicides with economic conditions [18, 20]. Results from developed countries regarding homicides were also mixed, with countercyclical oscillations [2, 6, 11, 42], procyclical oscillations [9], and no association [4, 8, 10, 12, 43]. Two factors can explain the relationship between homicides and business cycles. High unemployment rates and related problems of financial duress during recessions can induce stress or frustration leading to violent crime [44]. On the other hand, increase in alcohol consumption during economic expansions, added to expanding gun ownership, can increase the occurrence of lethal violent events [44]. Our finding seems to support the first hypothesis in the Latin American context.

Our analysis showed an acyclical oscillation of suicides in the total sample of Latin American cities. Most studies conducted in developed countries found an increase in suicides associated with economic downturns [45, 46]. It has been suggested that in Latin American countries the role of Catholicism could be responsible for the differential patterns between socioeconomic level and the risk of suicide with respect to developed countries [47]. So it is possible that the social cohesion promoted by Catholicism and other religions could act as a protective factor against the negative effects of recessions on suicide risk.

Our results for Mexico showed mostly acyclical oscillations of mortality. These results are different from those reported by González and Quast [21], who found procyclical associations in Mexico during 1993–2004 using state-level data. The discrepancy may be perhaps explained because our analysis used data from Mexican cities during the period 2005–2015 and just considered cities with more than 100,000 inhabitants. Taken at face value, our results compared with those of Gonzalez and Quast seem to suggest that in Mexico mortality changed from evolving mostly procyclically before 2005 to evolve mostly acyclically after 2005.

Our findings by tertiles of SES do not support the hypothesis of a different relationship between mortality and business cycles by level of development. Previous evidence from Mexico suggested procyclical mortality in areas of more developed or industrialized economy and countercyclical mortality in the rest of the country [22]. The hypothesis of economic expansions reducing mortality in areas of low SES but increasing it in areas of high SES does not seem compatible with our results, as most effects we found in the analysis stratified by tertiles of city SES reveal nonsignificant effects without any pattern in signs. One explanation for this could perhaps be the expansion of heterogeneous social policies that were applied in Latin American countries since the 2000s [48]. Territories with high investment in health and social protection programs could mitigate both procyclical [49] and countercyclical oscillations [18] of mortality.

What is the public health significance of our results? To answer that question requires assessing the size of the effects we found. We find the most intense procyclical oscillation of mortality in Peru, where one thousand US$ higher GDP per capita appears associated with around 60 extra deaths per 100,000 for the whole population or separately for men or women (Table 2). Given that according to our sample of Peruvian cities the population-weighted general mortality rate is 443 deaths per 100,000, the effect of 60 extra deaths per 1,000 extra US$ of income is an increase of 60/443 = 0.135 = 13.5% in total mortality. This is a quite large effect. In our general sample including all cities, the general mortality is 571 deaths per 100,000 and the estimated effect of 1,000 extra US$ of income is an increase of about 3 deaths per 100,000 (Table 2). That represents an increase of 3/571 = 0.005 = 0.5% in general mortality associated with 1,000 extra US$ of income. This is a quite smaller effect but still quite important from a demographic or public health point of view. Thus we found a wide range of positive effects of GDP on mortality, but always indicating substantial procyclical oscillation of mortality. The few instances of countercyclical mortality that we found are probably, with the exception of homicides, isolated cases due to the computation of multiple models.

Our study has several limitations. First, we used GDP per capita as business cycle indicator, while most of previous studies used unemployment and when GDP was used it was found a much worse predictor than unemployment of the changes in mortality. Unfortunately, unemployment data at the city-level is available for only a few urban areas in our sample. Furthermore, because of high frequency of informal employment, the unemployment rate may be a poor business cycle indicator in developing countries and Latin American countries in particular [17, 22]. Second, sub-national GDP per capita was used as business cycle indicator. This indicator assumes similar changes in GDP per capita across all cities in a sub-national territory (i.e., provinces in Argentina, states in Brazil or Mexico, etc.). Third, the relationship between business cycles and mortality can be contingent [17, 50], while we assume a constant relationship between both phenomena when we combine cities from different countries and, therefore, different periods of time. Fourth, the panel we used is very unbalanced with data corresponding to just one country before 2002. However, the sensitivity analysis restricting the data for Colombia to the 2002–2015 period did not, for the most part, alter the results found in the models with the entire sample of cities and years. Fifth, although various techniques were used to account for missing information, ill-defined deaths, and underreporting of deaths, they are imperfect, producing under-or over-correction of deaths. However, errors in variables do not tend to produce patterns but contrarily, tend to blur them, except if the errors are associated with the variables in the model. If, for instance registration of deaths significantly deteriorates in periods of economic downturn, that could explain in part the pattern of procyclical mortality. Sixth, we did not include race or ethnicity variables as independent variables related to the demographic structure of cities. Unfortunately, this type of information is available in few of the countries included in our sample.

Despite the limitations of this study, our results show that in our general sample and our samples of cities of ten Latin American countries, as well as in the samples of cities of Chile, Colombia, and Peru, and specific age-groups and causes of death, once long-term trends are taken into account, economic expansions are associated with an increase in mortality. Our results do not support the notion that countercyclical oscillations of mortality are proper of areas of low SES while procyclical oscillations of mortality are restricted to high SES levels. More studies with better data will be convenient to confirm the patterns observed in this paper in cities of these and other developing countries.

## Supplementary Material

Supplementary file

## Figures and Tables

**Table 1:** Descriptive statistics of the variables used in the models. Values weighted by the population size of each city (Salud Urbana en América Latina Project, ten countries of Latin America, 1990–2015).

Variable	Mean	Median	Stand. Dev.	Minimum	Maximum
Death rate per 100,000 population					
Total mortality	571	560	113	58	1,103
Female	491	479	108	46	1,045
Male	656	650	133	70	1,425
0–9 years	182	172	61	16	1,110
10–29 years	121	106	68	22	790
30–44 years	228	221	73	42	1,162
45–64 years	740	749	158	121	2031
65+ years	4,742	4,657	641	547	13,800
Cardiovascular diseases	161	152	48	14	384
Respiratory diseases	36	33	16	0	213
Malignant neoplasms	97	94	28	9	233
Diabetes mellitus	38	26	27	0	173
Respiratory infections	29	23	17	2	150
Infectious and parasitic diseases	27	25	12	2	252
Nutritional deficiencies	5	4	4	0	49
Road traffic injuries	17	16	8	0	75
Suicide	5	5	3	0	29
Homicide	34	24	37	0	453
Covariates					
GDP per capita^1^	14	12	10	1	148
Population 0–9 years (%)	17	17	3	10	31
Population 65+ years (%)	7	6	2	1	15
Masculinity index^2^	94	94	5	76	129
Female population 0–9 years (%)	16	16	3	10	31
Male population 0–9 years (%)	18	18	3	11	32
Female population 65+ years (%)	7	7	2	1	18
Male population 65+ years (%)	6	6	1	1	14

1 Thousand US$ at 2011 prices.

2 Men per 100 women in the population.

**Table 2:** Effects of one thousand US$ (at 2011 prices) of added GDP per capita on mortality (per 100,000 population), as estimated from regressions in which observations are weighted by the square root of the population size^1^ (Salud Urbana en América Latina Project, ten countries of Latin America, 1990–2015).

Dependent variable (death rate per 100,000 population)	All cities		Country													
			Argentina		Brazil		Chile		Colombia		Mexico		Peru	
	M1^2^	M2^3^	M1	M2	M1	M2	M1	M2	M1	M2	M1	M2	M1	M2
Total mortality	3.08^+^	2.83^*^	7.70	7.99	1.93	1.36	6.67^+^	7.01^*^	9.83^*^	7.16^+^	–6.10	–4.67	59.46^*^	57.55^*^
Female mortality	4.1^***^	3.95^***^	7.83	8.08	2.69^*^	2.50^*^	7.39^*^	7.57^*^	6.44^*^	4.98^*^	0.27	0.14	55.99^*^	54.10^*^
Male mortality	2.29	1.92	8.57	8.92	1.15	0.26	5.97	6.40^+^	13.25^*^	9.52	–12.62^+^	–9.49^+^	62.49^*^	60.59^*^
0–9 years	4.11^***^	2.50^***^	3.72	3.65	1.22	1.15	3.55^+^	3.20	10.45^**^	3.49	1.37	1.15	53.05^+^	52.73^+^
10–29 years	–1.46	–2.10	1.45	1.48	–1.61^+^	–1.85^*^	0.02	0.08	6.84^+^	5.08	–8.90^+^	–7.12^+^	9.74^+^	9.68^+^
30–44 years	–1.12	–1.78	–0.77	–0.71	–1.62	–1.69^+^	2.74	3.22	6.49	3.67	–9.07	–6.70	13.68	14.39
45–64 years	6.04^***^	5.14^***^	25.88^+^	25.97^*^	1.38	0.77	10.12^*^	10.32^*^	9.29^+^	6.25	2.47	1.72	32.01	33.95
65+ years	40.35^**^	34.35^***^	115.26	117.25	31.11^*^	26.46^*^	49.65	51.68	47.58	49.99^+^	1.95	1.23	375.41	385.22
Cardiovascular diseases	1.33^**^	1.21^***^	5.94	5.98	1.15^*^	1.09^*^	1.53	2.13^+^	2.69^*^	2.45^**^	0.06	–0.04	9.70^**^	9.39^**^
Respiratory diseases	0.03	0.22	–5.60^*^	–5.49^*^	0.13	–0.06	0.60^+^	0.51^+^	–0.00	0.08	–0.38^*^	–0.36^*^	12.15	11.89
Malignant neoplasms	0.52^*^	0.42^*^	2.48	2.50	0.08	–0.01	0.47	0.68	0.66	–0.08	0.12	0.10	8.32^+^	7.97^*^
Diabetes mellitus	0.41^***^	0.28^**^	1.77^+^	1.78^*^	–0.03	0.00	0.16	0.07	0.29	0.28^+^	–0.18	–0.32	0.00	–0.12
Respiratory infections	0.37^**^	0.46^***^	6.23^*^	6.30^*^	0.61^**^	0.56^**^	0.06	0.40	0.24	0.24	0.21	0.20	1.58	0.84
Infectious and parasitic diseases	0.15	0.21^+^	–6.66^*^	–6.61^*^	–0.18	–0.23	0.16	0.26	0.26	0.13	0.15	0.14	10.16^+^	9.90^+^
Nutritional deficiencies	0.06	0.04	0.03	0.04	–0.03	–0.02	0.27	0.29	0.10	0.19	0.21	0.20^+^	0.60	0.63
Road traffic injuries	0.68^***^	0.58^***^	–0.33	–0.32	–0.26	–0.37^*^	0.63^**^	0.47^**^	1.03^*^	1.06^*^	0.15	0.22	–1.11	–1.21
Suicide	0.07	0.07	0.80	0.78	0.06	0.05	0.40	0.48	0.07	0.05	0.16	0.13	–0.60^+^	–0.63^*^
Homicide	–1.73	–1.79^*^	0.54	0.54	–0.07	–0.12	–0.17	–0.24	2.87	1.22	–7.26^+^	–5.45^+^	0.25	0.21
Observations	4383		198		1806		252		910		979		184	

1 Regressions in which the dependent variable is an age-specific rate only include as demographic covariate an index of masculinity. All other regressions include the population proportions of children younger than 10 and elderly older than 64.

2 Model including fixed effects for city and year, and city-specific linear trends.

3 Model with series detrended with the Hodrick-Prescott filter with a smoothing parameter γ = 100.

*** p < *0.001*.

** p < *0.01*.

* p < *0.05*.

+ p < *0.10*.

**Table 3:** Effects of one thousand US$ (at 2011 prices) of added GDP per capita on mortality (per 100,000 population), as estimated from regressions in which observations are weighted by the square root of the population size^1^ (Salud Urbana en América Latina Project, ten countries of Latin America, 1990–2015).

Dependent variable (death rate per 100,000 population)	Tertiles of socioeconomic status (SES)^2^					
	First tertile (low SES)		Second tertile(medium SES)		Third tertile (high SES)	
	M1^3^	M2^4^	M1	M2	M1	M2
Total mortality	3.84	1.45	0.93	0.33	0.65	1.84
Female	3.49	1.71	4.70^***^	3.68^**^	1.32	2.33^+^
Male	4.51	1.30	–2.48	–2.55	0.05	1.52
0–9 years	5.13	3.14	2.08	0.95	1.75^*^	1.99^**^
10–29 years	1.17	0.76	–4.84	–7.08^*^	–1.07	–0.33
30–44 years	2.02	0.75	–5.57	–8.42^+^	–0.61	0.35
45–64 years	5.78	2.06	6.49^*^	4.77	1.95	3.81^**^
65+ years	50.16	0.36	44.25^**^	40.57^**^	12.14	23.50^*^
Cardiovascular diseases	0.66	–0.88	1.20^*^	0.82^+^	0.59	0.95
Respiratory diseases	–0.80	–0.23	0.09	0.13	0.14	0.27
Malignant neoplasms	0.86^*^	0.50	1.01^*^	0.72^+^	–0.08	–0.004
Diabetes mellitus	0.40	0.50	0.31	0.18	0.37^*^	0.22
Respiratory infections	–0.01	–0.21	0.35^*^	0.45^**^	0.22	0.34^+^
Infectious and parasitic diseases	–0.17	0.02	0.38^*^	0.45^*^	–0.28^+^	–0.16
Nutritional deficiencies	0.30	0.23	0.003	–0.03	–0.09	–0.02
Road traffic injuries	0.44	0.16	0.80^**^	0.76^**^	0.43^***^	0.48^***^
Suicide	0.13	0.09	0.07	0.10	0.07	0.06
Homicide	0.74	–0.01	–4.73	–4.58^*^	–0.67	–0.30

1 Regressions in which the dependent variable is an age-specific rate only include as demographic covariate an index of masculinity. All other regressions include the population proportions of children younger than 10 and elderly older than 64.

2 Urban areas (n = 340) classified according to GDP per capita of 2014. Observations (years-city) = 4,383.

3 Model including fixed effects for city and year, and city-specific linear trends.

4 Model with series detrended with the Hodrick-Prescott filter with a smoothing parameter γ = 100.

*** p < *0.001*.

** p < *0.01*.

* p < *0.05*.

+ p < *0.10*.
