# Supplementary material for: Are Wealthier Times Healthier in Cities? Economic Fluctuations and Mortality in Urban Areas of Latin America
Source: Int J Public Health. Author manuscript; Available in PMC 2022 Jan 2. (PMC8696345; doi:10.3389/ijph.2021.1604318)
Supplement: Supplementary file [file EMS140653-supplement-Supplementary_file.docx]

**Supplementary material**

Table A1. Countries, sample periods and cities included in the models (Salud Urbana en América Latina Project, ten countries of Latin America, 1990-2015).

| Country | Time frame | Number of cities |
| --- | --- | --- |
| Argentina | 2010-2015 | 33 |
| Brazil | 2002-2015 | 129 |
| Chile | 2004-2015 | 21 |
| Colombia | 1990-2015 | 35 |
| Costa Rica | 2010-2015 | 1 |
| Guatemala | 2009-2015 | 3 |
| Mexico | 2005-2015 | 89 |
| Panama | 2012-2015 | 3 |
| Peru | 2008-2015 | 23 |
| El Salvador | 2010-2014 | 3 |

Table A2. ICD codes included for each cause of death. Fourth codes in brackets (Salud Urbana en América Latina Project, ten countries of Latin America, 1990-2015).

| **Cause of death** | **ICD-9 codes** | **ICD-10 codes** |
| --- | --- | --- |
| Infectious and parasitic diseases | 001-139, 279 [5], 320-323, 614-616, 771 [3] | A00-B99, G00-G04, G14, N70-N73, U06 [9] |
| Cardiovascular diseases | 390-459 | I00-I99 |
| Diabetes mellitus | 249-250 | E10-E14 |
| Respiratory infections | 381-382, 460-466, 480-487 | H65-H66, J00-J06, J09-J18, J20-J22 |
| Malignant neoplasms | 140-208, 209 [0-3] | C00-C97 |
| Nutritional deficiencies | 243, 260-269, 280-281, 285 [9] | D50-D53, D64.9, E00-E02, E40-E46, E50-E64 |
| Respiratory diseases | 470-478, 490-519 | J30-J98 |
| Road traffic injuries | 810-819, 826-829, 929 [0] | V01-V06 [1-9], V09 [2-3], V10-V14 [3-9], V15-V19 [4-9], V20-V28 [3-9], V29-V79 [4-9], V80 [3-5], V81 [1], V82 [1, 8, 9], V83-V86 [0-3], V87, V89 [2, 3, 9], V99, Y85 [0] |
| Suicide | 950-959 | X60-X84, Y87 |
| Homicide | 800-807, 820-825, 830-848, 870-879, 911, 912, 915, 924-949, 960-969 | X85-X99, Y00-Y09, Y35, Y87 [1] |

Table A3. Effects of one lag of one thousand US$ (at 2011 prices) of added GDP per capita on mortality (per 100,000 population), as estimated from regressions in which observations are weighted by the square root of the population size^1^ (Salud Urbana en América Latina Project, ten countries of Latin America, 1990-2015).

| Dependent variable (death rate per 100,000 population) | All cities | | | Country | | | | | | | | | | | | | | | |  |
| --- | --- | --- | --- | --- | --- | --- | --- | --- | --- | --- | --- | --- | --- | --- | --- | --- | --- | --- | --- | --- |
|  |  |  |  | Argentina | | | Brazil | | Chile | | Colombia | | | Mexico | | | Peru | | |  |
|  | M3^2^ | M4^3^ | M3 | | M4 | M3 | | M4 | M3 | M4 | | M3 | M4 | | M3 | M4 | | M3 | M4 | |
| Total mortality | 2.37 | 1.48 | 2.37 | | -13.23 | 1.12 | | .22 | 5.41* | 5.48** | | 8.81* | 5.73^+^ | | -8.29^+^ | -7.02^+^ | | 12.21 | 9.42 | |
| Female mortality | 3.47** | 2.60*** | 18.83 | | -3.64 | .94 | | .56 | 6.48** | 6.35** | | 6.09^+^ | 4.33^+^ | | -.22 | -.29 | | 18.25 | 13.75 | |
| Male mortality | 1.51 | .42 | -9.54 | | -22.39 | 1.14 | | -.24 | 4.35 | 4.57^+^ | | 11.34^+^ | 6.96 | | -16.53^+^ | -13.61^+^ | | 6.27 | 5.34 | |
| 0-9 years | 4.57*** | 2.65** | 5.20 | | .58 | 2.05^+^ | | 2.18* | 2.04^+^ | 1.65 | | 10.77** | 4.97 | | 1.07 | .74 | | 2.96 | 3.96 | |
| 10-29 years | -1.48 | -1.82 | .83 | | -.24 | .14 | | .16 | 2.07 | 2.34^+^ | | 5.15 | 2.98 | | -11.90* | -10.62* | | -.34 | .18 | |
| 30-44 years | -1.42 | -1.91 | -6.90 | | -3.08 | 1.04 | | 1.11 | 1.62^+^ | 1.87^+^ | | 5.22 | 2.59 | | -13.98^+^ | -12.63* | | 7.48 | 10.66 | |
| 45-64 years | 5.77** | 4.12*** | 17.16 | | -3.01 | 1.44 | | .57 | 8.73* | 8.95* | | 9.91* | 5.84^+^ | | .96 | .14 | | 11.22 | 18.13 | |
| 65+ years | 28.17* | 14.87* | -123.97^+^ | | -154.30* | -9.52 | | -18.96* | 34.72^+^ | 35.93* | | 55.19 | 42.96^+^ | | 5.22 | 5.11 | | 140.58 | 180.70 | |
| Cardiovascular diseases | 1.06** | .52^+^ | 6.43 | | -7.75* | -.08 | | -.31 | 1.36 | 1.93^+^ | | 2.67* | 1.95^+^ | | -.18 | -.28 | | .17 | -.28 | |
| Respiratory diseases | .27 | .41** | -4.74* | | -2.19 | .55^+^ | | .40^+^ | .14 | -.01 | | .12 | .14 | | -.27 | -.21 | | 3.84 | 3.57 | |
| Malignant neoplasms | .15 | -.02 | 2.86 | | 1.26 | -.10 | | -.16 | -.23 | -.23 | | .55 | -.10 | | .07 | .04 | | -1.27 | -1.74 | |
| Diabetes mellitus | .50*** | .35** | 1.76 | | -.21 | -.02 | | -.01 | .22 | .21 | | .23 | .04 | | .73* | .76** | | .89 | .50 | |
| Respiratory infections | .26* | .30** | 3.02 | | -2.89* | .34 | | .28 | .04 | .38 | | .21 | .29 | | .08 | .06 | | 1.28 | -.99 | |
| Infectious and parasitic diseases | .10 | .13 | -8.52** | | -.10 | -.31^+^ | | -.35^+^ | .20 | .32 | | .42 | .43 | | .25 | .26 | | 6.89 | 6.69 | |
| Nutritional deficiencies | .06 | .07^+^ | -.57 | | -.31 | -.10 | | -.09 | .35^+^ | .35* | | .15 | .29* | | .10 | .07 | | -.38 | -.27 | |
| Road traffic injuries | .65*** | .43*** | -.13 | | -.18 | .46^+^ | | .30 | .93** | .85* | | .95* | .99** | | .18 | .19 | | .82 | .44 | |
| Suicide | .03 | .01 | 1.53 | | .31 | -.01 | | -.02 | -.12 | -.13 | | .01 | -.06 | | .12 | .10 | | .29 | .16 | |
| Homicide | -1.71 | -1.64 | .88 | | -.05 | .64 | | .53^+^ | .34 | .34 | | 2.32 | .98 | | -9.51^+^ | -7.94^+^ | | -.99^+^ | -1.10* | |

^1^ Regressions in which the dependent variable is an age-specific rate only include an index of masculinity. All other regressions also include as covariates the population proportions of children younger than 10 years and elderly older than 64. Regressions in which the dependent variable is an sex-specific rate only included the population proportions of children younger than 10 years and elderly older than 64.

^2^ Fixed effects for city and year, and city-specific linear trends.

^3^ Series detrended with the Hodrick-Prescott filter with a smoothing parameter γ = 100.

^∗∗∗^*P* < 0.001, ^∗∗^*P* < 0.01, ^∗^*P* < 0.05, ^+^*P* < 0.10

Table A4. Effects of one thousand US$ (at 2011 prices) of added GDP per capita on mortality (per 100,000 population), as estimated from regressions in which observations are weighted by the square root of the population size^1, 2^ (Salud Urbana en América Latina Project, ten countries of Latin America, 1990-2015).

| Dependent variable (death rate per 100,000 population) | M1^3^ | M2^4^ |
| --- | --- | --- |
| Total mortality | 1.57 | 2.05 |
| Female mortality | 3.73*** | 3.71*** |
| Male mortality | -.33 | .71 |
| 0-9 years | 1.98** | 2.10** |
| 10-29 years | -4.07* | -3.01* |
| 30-44 years | -3.77 | -2.64 |
| 45-64 years | 5.05** | 5.11*** |
| 65+ years | 35.49*** | 31.24*** |
| Cardiovascular diseases | 1.13** | 1.04** |
| Respiratory diseases | .10 | .20 |
| Malignant neoplasms | .46* | .45* |
| Diabetes mellitus | .42** | .27* |
| Respiratory infections | .40** | .45*** |
| Infectious and parasitic diseases | .18 | .18 |
| Nutritional deficiencies | .06 | .03 |
| Road traffic injuries | .49*** | .49*** |
| Suicide | .08 | .07 |
| Homicide | -2.87* | -2.17* |

^1^ Regressions in which the dependent variable is an age-specific rate only include as demographic covariate an index of masculinity. All other regressions include the population proportions of children younger than 10 and elderly older than 64.

^2^ Regressions include only years 2002-2015 in Colombia.

^3^ Model including fixed effects for city and year, and city-specific linear trends.

^4^ Model with series detrended with the Hodrick-Prescott filter with a smoothing parameter γ = 100.

^∗∗∗^*P* < 0.001, ^∗∗^*P* < 0.01, ^∗^*P* < 0.05, ^+^*P* < 0.10

Table A5. Effects of one thousand US$ (at 2011 prices) of added GDP per capita on mortality (per 100,000 population), as estimated from regressions in which observations are weighted by the square root of the population size^1^ (Salud Urbana en América Latina Project, ten countries of Latin America, 1990-2015).

| Dependent variable (death rate per 100,000 population) | Excluding cities of Costa Rica, El Salvador and Panama | | Excluding cities of Brazil | |
| --- | --- | --- | --- | --- |
|  | M1^2^ | M2^3^ | M1 | M2 |
| Total mortality | 3.06^+^ | 2.81* | 4.56 | 3.44 |
| Female mortality | 4.14*** | 3.96*** | 5.00** | 4.53** |
| Male mortality | 2.26 | 1.87 | 4.37 | 2.64 |
| 0-9 years | 4.11*** | 2.52*** | 6.52*** | 3.41** |
| 10-29 years | -1.47 | -2.12 | .11 | -1.34 |
| 30-44 years | -1.14 | -1.82 | .20 | -1.28 |
| 45-64 years | 6.02*** | 5.14*** | 7.42** | 5.89* |
| 65+ years | 40.26** | 34.35*** | 44.71* | 46.18** |
| Cardiovascular diseases | 1.32** | 1.22*** | 1.50** | 1.30** |
| Respiratory diseases | .032 | .22 | .073 | .16 |
| Malignant neoplasms | .51* | .42* | .66* | .59* |
| Diabetes mellitus | .41*** | .28** | .19 | .14 |
| Respiratory infections | .37** | .46*** | .30^+^ | .36* |
| Infectious and parasitic diseases | .15 | .20^+^ | .36^+^ | .40* |
| Nutritional deficiencies | .06 | .04 | .15 | .15* |
| Road traffic injuries | .67*** | .58*** | .76** | .72*** |
| Suicide | .07 | .07 | .12^+^ | .11 |
| Homicide | -1.74 | -1.80* | -1.24 | -1.98 |

^1^ Regressions in which the dependent variable is an age-specific rate only include an index of masculinity. All other regressions also include as covariates the population proportions of children younger than 10 years and elderly older than 64. Regressions in which the dependent variable is an sex-specific rate only included the population proportions of children younger than 10 years and elderly older than 64.

^2^ Fixed effects for city and year, and city-specific linear trends.

^3^ Series detrended with the Hodrick-Prescott filter with a smoothing parameter γ = 100.

^∗∗∗^*P* < 0.001, ^∗∗^*P* < 0.01, ^∗^*P* < 0.05, ^+^*P* < 0.10
